# *Legionella* Occurrence beyond Cooling Towers and Premise Plumbing

**DOI:** 10.3390/microorganisms9122543

**Published:** 2021-12-09

**Authors:** David Otto Schwake, Absar Alum, Morteza Abbaszadegan

**Affiliations:** 1 Department of Natural Sciences, Middle Georgia State University, 100 University Pkwy, Macon, GA 31206, USA; otto.schwake@mga.edu; 2 School of Sustainable Engineering and the Built Environment, Arizona State University, Tempe, AZ 85287, USA; alum@asu.edu

**Keywords:** *Legionella pneumophila*, detection methods, freshwater, groundwater, saltwater, drinking water

## Abstract

*Legionella* is an environmental pathogen that is responsible for respiratory disease and is a common causative agent of water-related outbreaks. Due to their ability to survive in a broad range of environments, transmission of legionellosis is possible from a variety of sources. Unfortunately, a disproportionate amount of research that is devoted to studying the occurrence of *Legionella* in environmental reservoirs is aimed toward cooling towers and premise plumbing. As confirmed transmission of *Legionella* has been linked to many other sources, an over-emphasis on the most common sources may be detrimental to increasing understanding of the spread of legionellosis. This review aims to address this issue by cataloguing studies which have examined the occurrence of *Legionella* in less commonly investigated environments. By summarizing and discussing reports of *Legionella* in fresh water, ground water, saltwater, and distribution system drinking water, future environmental and public health researchers will have a resource to aid in investigating these pathogens in relevant sources.

## 1. Introduction

Since their discovery as the causative agent of Legionnaire’s disease in 1976 [1], bacteria of the *Legionella* genus have become a major source of drinking water-related disease outbreaks [2]. Naturally occurring in water [3] and soil [4], *Legionella* possess traits that enable their survival in a wide variety of environmental conditions [5], including protozoan host parasitization [6]. Though numerous transmission sources have been implicated in the spread of legionellosis, transmission is thought to occur primarily through the inhalation of cells, commonly via aerosolized water from engineered systems [7]. Therefore, identifying potential transmission sources is a key step in learning more about the infectious risks of legionellosis [8]. Despite the known presence of *Legionella* in a multitude of environments, to date, a large portion of legionellosis epidemiological studies and monitoring programs have been focused on transmission that is linked to premise plumbing or cooling towers [9,10,11,12,13,14]. As these other reservoirs have lower reported contribution to human disease, it could be inferred that less studied sources of contamination are unimportant from a public health perspective. However, legionellosis cases have been traced to seemingly uncommon routes of transmission and the historical over-emphasis on cooling towers and tap-water may, ironically, even be partially responsible for under-reporting of legionellosis that is attributed to other contaminated sources [15].

The goal of this review is to address the potentially problematic de-emphasis of poorly understood environmental reservoirs for *Legionella* by summarizing their occurrence in the following sources that are related to drinking water: fresh water, ground water, saltwater, and distribution system drinking water. While outlining the reports of contamination in these environments, relevant conclusions that are posed by the individual studies will be discussed. In addition, certain methodological aspects from the various studies will be listed, as well as key results, such as positivity rates, concentration, and the detected species. The information that is provided may prove useful in further investigations on *Legionella* presence and concentrations in a variety of sources and in the determination of potential public health ramifications due to their occurrence. 

## 2. Surface Freshwater

One of the first identified natural environmental sources for *Legionella*, surface freshwater, may also be one of the more relevant with regard to public health. The potential for human exposure to *Legionella* in natural surface water can come in many forms including directly through recreational water activities or more incidentally via aerosolization from bodies of water. Perhaps most important is the fact that surface water sources are often used to supply drinking water treatment plants and other engineered water systems. While conventional water treatment effectively reduces levels of fecally-derived microbes to relatively safe levels, *Legionella’s* ubiquity in surface water and the ability for regrowth and long-term survival in drinking water systems [16] can present risks that are not common with water-borne gastrointestinal pathogens. The summarized results from studies that are discussed in this section are listed in Table 1.

## 3. River Water

Shortly after the first reported cases of Legionnaires’ disease, environmental investigation determined rivers to be an environmental reservoir. An early study documented a near-constant presence of *L. pneumophila* in the littoral zones of lakes in the eastern United States [17]. Measurement by direct fluorescent antibody microscopy revealed >93% (14/15) positivity in samples from seven rivers. As the study was performed during the initial years of *Legionella* research, cross-reactivity was a concern for antibodies that were used, potentially impacting the high detection rates that were observed. 

Cold temperature freshwater sources (even at <20 °C) have the potential to contain *Legionella*. In the Netherlands, 100% of river water near freezing temperatures of 3–4 °C were found to have to have a wide variety of microbial species, including *L. pneumophila* [19]. Perhaps even more unexpected, relatively high concentrations up to 2.5 × 10^3^ cells/mL were recorded. It was speculated that the *Legionella* that were detected were survivors from the moderate summer or originated from wastewater discharge.

While pristine freshwater rivers with low levels of human contact may pose no direct legionellosis transmission risk, they still represent important natural reservoirs for *Legionella*. Water that was sampled from the Rio Branco River in the southern coast of Sao Paulo state, Brazil, was found to contain *L. pneumophila* [20]. Concentrated superficial water samples produced no culturable isolates and only the single species, in contrast to anthropogenically polluted downstream waters suggesting that there was contamination from a common source. The lower diversity and concentrations that were found in pristine sections of the same river could be common for similar natural environments. 

Examination of natural waters can lead to the identification of novel *Legionella* species, including potential human pathogens. Multiple independent samples from the Elbe River in Germany contained culturable isolates of a previously unidentified species, *L. dresdenensis* [25]. Phenotypically, it is similar to both *L. rubrilucens* and *L. pneumophila* but the new species had a unique serotype profile and a demonstrated capability of invading and replicating within amoeba hosts. The routine water sampling also produced several strains of *L. pneumophila*.

Anthropogenic pollution can often lead to elevated contamination of *Legionella* in rivers. An environmental study to investigate the source of an *L. pneumophila* serogroup 1 outbreak in Norway discovered high levels downstream of an outlet for a biological treatment plant along the Glomma River [26]. Concentrations decreased with distance from the plant outlet yet remained high at 40 colony forming unit (CFU)/mL up to 1.6 km downstream of the plant, demonstrating a long-reaching effect of the discharge.

While some studies have demonstrated the impact of anthropogenic waste and seasonality on *Legionella* contamination in natural bodies of water, this is not always the case. Samples from the Tech River in Southern France upstream and downstream of discharge from thermal baths or a wastewater treatment plant revealed a significant increase in *Legionella* in only one of three sampling sites [27]. In addition, the concentrations remained stable over different seasons, potentially due to small shifts in the water temperature of 0–6 °C. High levels of contamination naturally occurring in the river may have impacted the results, with 100% of samples containing *Legionella*, with concentrations as high as 900 genomic units (GU)/mL.

High levels of *Legionella* in source water can lead to unsafe levels in drinking water when combined with insufficient treatment. Sampling of sites along the Jiulong River, Fujian province, China, revealed high levels of *L. pneumophila*, reaching up to 2.5 × 10^4^ GU/mL [39]; all of the samples (16/16) tested positive. A nearby water treatment plant only demonstrated slightly greater than 1 log reduction in concentrations in treated water, presenting a scenario with a potential public health hazard.

The potential for water source biofilms to serve as habitats for *Legionella* was demonstrated in samples that were collected from the Yangtze River in Hubei province, China [31]. 100% (6/6) of biofilm samples from two branches, one natural river and one canal, contained *Legionella* and multiple species of amoebae. Although the concentrations were low relative to other bacteria, *Legionella* was found in all of the samples. 

Although growth of *Legionella* is typically associated with parasitization of eukaryotic hosts, the presence of commonly associated host amoebae is not necessary for high levels of contamination. Water samples from the Puzih River in Taiwan demonstrated high frequencies of occurrence, yet amoebae were rarely detected [35]. *Legionella* were present in 63.1% (41/65) of the samples, despite <9% containing *Hartmanella*, *Naegleria*, or *Acanthamoeba*. The measured concentrations up to 10^3^ GU/mL suggest alternative host organisms or extracellular replication allowed for the high levels that were observed in the river.

*Legionella* contamination in environmental sources is often linked to seasonality, with a higher frequency of detection typically observed in warmer months, although this is not always the case. A survey of five lakes and rivers in differing climate zones of South Korea demonstrated ubiquity of *L. pneumophila*, with positive PCR results from 14/100 samples [4]. While all of the sites had detectable *L. pneumophila* in at least one sample, higher positivity was observed during two sampling periods, both in winter. This atypical seasonality pattern throughout a variety of climates shows that other environmental factors beyond temperature play a role in *Legionella* populations.

## 4. Lake Water

Due to more stagnant conditions and potential for increased concentrations of nutrients, lakes may serve as more ideal natural habitats for *Legionella* than rivers. In an early environmental study of freshwater habitats, a large number of lakes and ponds in the United States (some thermally altered) were found to contain *L. pneumophila* [17]. More than 99% (767/771) of the samples from 55/56 sites tested positive by molecular analysis, despite the wide range of water quality parameters, including temperature (5.7–63 °C), pH (5.5–8.1), and oxygen concentration (0.3–8.2 mg/L). These results helped established the concept of ubiquity for *Legionella* in a wide variety of freshwater environments.

With the development of specific culture media, relevant data in the form of viable, culturable *Legionella* has been obtainable from environmental samples. Culturable *Legionella* were found in water from lakes and reservoirs in southern California, United States [18]. Although all of the samples tested positive by molecular or immunological assays, cultured isolates were only obtained from 25% (2/8) of the samples. Regardless of the lowered concentration and positivity that is often experienced with culture-based techniques, quantification of only living bacteria is an important advantage of this method.

Natural disasters often lead to increases in infectious disease rates, with hurricanes and floods often causing water pollution and civil infrastructure damage. An ecological study on the microbial water quality of Lake Pontchartrain, Louisiana, United States, following the effects of Hurricane Katrina revealed frequent *Legionella* contamination [21]. More than 72% (35/48) of the samples that were collected from the lake during fall, winter, and spring were positive, with lowered detection rates in winter suggesting seasonality. Despite frequent contamination of *Legionella* spp. and anthropogenic discharge into the lake, only a single sample was found to contain *L. pneumophila*. 

While microbial life is ubiquitous in lake environments, the presence of thermotolerant bacteria in Antarctic habitats could be considered unexpected. *Legionella* were detected in 0 °C water samples that were collected in summer from two pristine lakes in King George Island, Antarctica [22]. Culture-based techniques successfully isolated a single *L. pneumophila* colony, with multiple species that were detected via PCR in both lakes. The presence of a diverse *Legionella* population in this setting was attributed to high concentrations of metal ions and the presence of protozoan host organisms.

A wide variety of *Legionella* have been found in lakes and other freshwater bodies, yet certain species are infrequently observed in these environments. In a rare observation in freshwater, *L. longbeachae* was among the species that were detected in samples from Lake Taihu, China [29]. The shallowness of the lake, which has an average depth of 1.9 m, may have contributed to the occurrence of this typically soil-associated species. Transient amoebae populations may have also played a role as intracellular *Legionella* were nearly twice as common as those not associated with a host. 

Even with common occurrence, higher concentrations of *Legionella* in lakes may be caused by specific environmental conditions. During an environmental study to assess the impact of seasonal effects on the presence and species in Taiwanese reservoirs, extremely high concentrations of up to 1.6 × 10^6^ cells/mL and 7.35 × 10^8^ cells/mL were measured [36,40]. Although positivity was the highest in fall, the concentrations tended to peak in summer, with warmer regions in the south having more stable populations. Correlations with chlorophyll, dissolved oxygen, and conductivity were also reported, potentially describing the conditions that are responsible for the levels that were detected.

Often associated with heated engineered systems, *Legionella* is a diverse genus with members that are capable of surviving in a variety of stressful environments. The detection of *Legionella* spp. via PCR in 7/19 (36.8%) of samples from glacier lakes in Antarctica demonstrates this point [38]. Similar community structure of *Legionella* within geographically distant sites, as well as positive detection in one hypersaline lake, highlight the adaptability of specific members of the genus to extreme conditions.

## 5. Rainwater

Rainwater harvesting has been implemented for thousands of years [41], however, with rising global water scarcity, this technology has seen increasing use in recent history. Although often noted as being of superior quality to poorly treated drinking water from conventional sources, the potential for gastrointestinal disease that is linked to rainwater has been established [42]. Unfortunately, far fewer investigations have been performed to examine this source for pathogens that are capable of causing acute respiratory disease such as *Legionella*.

Environmental sampling on water systems utilizing rainwater was performed in a Legionnaires’ disease outbreak investigation in Auckland, New Zealand [23]. Out of 48 homes that were sampled, 7 containing household rainwater collection systems were positive for *Legionella* with high concentrations of 3 × 10^2^ CFU/mL detected in a rainwater storage water tank. A rainwater-sourced water blaster at a local marina was also found to be contaminated with *L. pneumophila* serogroup 1, demonstrating an obvious route for transmission.

Rainwater that is harvested for potable use may not be the only form that is capable of legionellosis transmission, as rainwater puddles, particularly on roads, may pose a public health risk for drivers and pedestrians. *L. pneumophila* prevalence was detected in rain puddles on asphalt roads in Tokyo, Japan [24]. 1/10 rainwater samples were positive, and some puddles contained greater than 10 CFU/mL. A positive correlation between contamination and temperature was also noted, indicating a potentially increased risk for transmission from this source in warmer climates.

In another study, examination of 72 rainwater collection tanks from houses in 18 suburbs in Queensland, Australia demonstrated *Legionella* contamination [32]. Samples that were taken shortly after rains were analyzed by qPCR for the presence of *L. pneumophila*, with 6% of tanks being positive, with concentrations of 16–100 GU/mL. The large numbers of positive samples that were collected when water systems should have held the freshest and least stagnant water demonstrates the potential for contamination of these systems.

The presence of non-*pneumophila* species may also pose a health risk in drinking water systems. Metagenomic analysis of roof harvested rainwater collection tanks in Cape Province, South Africa, were shown to be frequently contaminated with several *Legionella* species [33]. Pyrosequencing identified eight known species in samples from 6/7 rainwater collection systems, including *L. longbeachae*, and *Legionella* were the most prevalent water pathogen that was identified. The fact that contamination was more extensive in harvested rainwater than in a local river highlights the need for proper water quality regardless of the source.

Although found in cold weather climates, it is reasonable to assume that low air temperatures could have an impact on the presence of *Legionella* in rainwater. Regardless, rainwater puddles sampled in the Netherlands were found to be contaminated [34]. Positivity was low (albeit with culture-based assays), with 3/77 samples that were positive for *Legionella*, including *L. pneumophila*. High ambient temperature of 20.3 °C may have contributed to these results, although it should be noted that all of the temperatures that were measured the day of sampling and in the preceding 14 days (6.9–20.3 °C) were near or below the typically minimum range for *Legionella* growth [43].

To date, most of the studies examining the occurrence of *Legionella* in natural water sources have been conducted on surface freshwater samples. Even though the majority of this research has documented generally low concentrations, vastly differing study-specific results have been produced. These highly variable reports on occurrence in freshwater could be, in part, due to a number of environmental factors differing between the study sites. Due to this apparent importance of habitat on the presence and concentration of *Legionella*, increasing knowledge on these relevant natural reservoirs under different conditions will continue to be important to gain a better understanding of the ecology of these pathogens.

## 6. Ground Freshwater

Similar to surface water, groundwater is often used as a source for drinking and recreational purposes. Typically, lower levels of water-borne pathogens that are present in non-contaminated sources, however, often leads to the treatment of groundwater being less substantial than for surface water. While these decreased levels of harmful microbes tend to hold true for gastrointestinal pathogens, *Legionella* have been shown to naturally inhabit even deep groundwater, albeit with lower positivity and concentrations compared to neighboring surface water. Potentially due to the reduced treatment/awareness and environmental factors such as natural heat, groundwater serves as a relatively common source of legionellosis outbreaks [44].

Concerning *Legionella* occurrence, groundwater is similar to surface freshwater, yet different in many ways. While surface water outside of tropical climates is commonly too cold to facilitate *Legionella* growth, groundwater can be heated by geothermal activity to temperatures that are optimal for growth. Although groundwater often lacks the potential for direct human contact, natural springs and artificial spas utilizing groundwater have long been established as habitats for *Legionella* [45] and routes of legionellosis transmission [46], demonstrating the importance of this source that is often neglected from a public health perspective. The summarized results from studies that discussed in this section are listed in Table 2.

## 7. Wells

Groundwater sources supplying water for potable purposes have long demonstrated potential for contamination. In the first large ecological study of this source, frequent contamination was detected in groundwater well samples from 16 states in the United States [18]. Nearly 95% of the samples from 29 public wells tested positive for a number of *Legionella* species. Although no culture positives were achieved and no samples contained *L. pneumophila* specifically, the high frequency of occurrence and concentrations > 40 cells/mL of other species demonstrated the potential for risk that is associated with this source.

The presence of PCR inhibitors in groundwater samples has been shown to drastically impact the accuracy of this detection method. *Legionella* were detected at high frequencies in both water (83%) and biofilm (75%) samples from two sites in the United States [48]. Decreased molecular detection was reported for both samples types when compared to cultivation, with 1/3 of samples exhibiting high levels of PCR inhibition. Heat enrichment of the samples at 35 °C was found to increase the accuracy of PCR detection.

Analysis of biofilms in groundwater wells have demonstrated frequent contamination, occasionally with surprising results. Biofilms from water meters, end caps, pipes, and coupons from wells in multiple states in the United States and provinces in Canada were found to contain up to 1.2 × 10^2^ CFU/cm^2^, including *L. pneumophila* [49]. Wells that were sampled were not under the direct influence of surface water and received no recycled water, ruling out these sources as points of contamination. Culture-based techniques demonstrated increased levels of detection in water samples compared to molecular detection (44% vs. 26%) and water had higher positivity than biofilm (58% vs. 34%). 

Hydrothermal groundwater that is heated to temperatures within the growth range of *Legionella* may have increased level of contamination. Groundwater samples from a series of boreholes in two geographically separated areas in central Portugal demonstrated a high frequency of occurrence [50]. Geothermal activity heated both areas (one containing artesian wells, the other a natural spring) to temperatures from 35–48 °C. Samples that were collected over a series of seven years revealed culturable *Legionella*, with >58% (68/116) of the samples tested positive. Several trends were observed, including a lack of positive samples during (but not after) chlorination, *L. pneumophila* presence in 100% of samples from one well, and no detection during the first year of operation of a new well. 

Due to the metabolic requirement for oxygen, lower levels of *Legionella* are often noted in anaerobic waters. Molecular analysis of anaerobic and aerobic groundwater in the Netherlands revealed an increased frequency and concentrations in the latter [19]. Positivity was over double at 88%, while the maximum levels that were measured of 25 cells/mL were an order higher for aerobic water. A diverse array of 12 species were identified, *L. worsleiensis* being the most common, potentially due to high variable levels of oxygen and high concentrations of metals in the samples. Significantly lower diversity was reported in a similar study on groundwater supplies in the Netherlands [52], with the trend of higher detection in oxygen-rich water maintained.

Although the highest levels of *Legionella* in groundwater have been measured in heated sources, cold water wells have also demonstrated contamination. Groundwater samples from wells in Eastern Poland demonstrated 62.5% (10/16) positivity by molecular detection [53]. The samples were obtained directly from wells or from unheated and untreated private water supply taps on farms. Significantly lower positivity by culturing (6.3%) could have been related to the low water temperatures reducing the cultivability of the *Legionella*.

Shallow water tables present many unique conditions for groundwater including being under the direct influence of surface water and an increased potential for intrusion of microbes. Microarray analysis was performed on groundwater samples from wells in the Kathmandu Valley, Nepal to determine the occurrence of pathogens [57]. *Legionella* were among the 26 genera of pathogens that were detected and were present in 73% of samples that were collected from wells 4.6–12.2 m deep. In addition to the shallow depths of the wells, relatively warm water (up to 24 °C) and the effects of the monsoon season may have contributed to the high levels of contamination. 

Water temperature and seasonality are often reported as being both correlated to *Legionella* contamination in groundwater sources or having little impact. Other environmental factors such as aquifer composition may play a larger role, as suggested in a study of Italian wells [61]. Culturable *Legionella* was detected in 31/145 wells, with PCR on the remaining 114 detecting DNA markers in 37 of the remaining wells. Water temperature was only weakly correlated with concentrations, but samples that were sourced from wells in porous as opposed to karst-fissured aquifers had higher levels, demonstrating an example of the complex interplay of environmental factors on *Legionella* populations.

## 8. Springs

Certain sources of groundwater can pose a significant risk of direct human exposure, with hot springs being popular recreational bathing sites in certain regions. The presence of *Legionella* in this source has been reported for many years [47] with environmental sampling within a French hot spring spa detecting 15 culturable species or serogroups, including *L. pneumophila*. Antibody titers of patients and therapists at the spa were highest for the dominant serogroup that was cultured. Renewed interest in bathing springs that are implicated in legionellosis transmission has prompted multiple recent studies, most in Asian countries. 

*Legionella* were detected in weak alkaline carbonate spring water both from the groundwater source and samples that were collected in Taiwanese recreational areas [51]. A contamination rate of 33% was observed in hot springs/streams that were not impacted by humans and tubs in a bathing facility. Amoeba co-culturing greatly increased the detection rates in certain samples. *Legionella* were also detected in sodium bicarbonate and sulfur springs in Taiwan at similar levels [40]). Here, 38% of the samples that were collected from three springs tested positive for *Legionella* within host amoebae. *H. vermiformis* was the most commonly identified host and no free-living *L. pneumophila* were detected. 

Unattended natural hot springs, known as noyu, are popular recreational water sites in Japan. Sampling of noyu from 11 prefectures revealed frequent *Legionella* contamination [54]. While concentrations were low, with a maximum of 3 CFU/mL, culturable *Legionella* were detected in 37% of the samples and 73% of prefectures. *L. pneumophila*, including serogroup 1, was by far the most common species, present in 87.5% (14/16) of the samples. The 33–41 °C sample temperatures may have contributed to the frequent occurrence detected. 

Like many recreational bathing waters, hot spring water is often untreated, even when feeding a bathing facility. An environmental study to evaluate the frequency of *L. pneumophila* in Tunisian hot springs recorded high levels of contamination in therapeutic spas that were supplied by untreated deep spring thermal waters [55]. More than 70% (54/77) of the samples that were collected from spring outlets and facilities tested positive by molecular methods at a high concentration of 420 GU/mL, with significantly lower positivity and levels determined via culture-based techniques. Experiments demonstrated an *L. pneumophila* isolate to be more resistant to heat shock than a clinical strain; unsurprising given the 48–66 °C sample temperatures that were recorded.

Elevated temperatures and other factors that are associated with bathing facilities may lead to extreme levels of contamination in hot springs. *Legionella* were detected in samples from a resort in Beijing via culture-based techniques, quantitative PCR, and ethidium-monoazide quantitative PCR [56]. High positives (74–100%) were reported for each detection method, with all 121 samples testing positive by molecular techniques. The measured concentrations were particularly high for the sites that were sampled, with maximum levels reaching 1.5 × 10^3^ GU/mL.

The correlation between *Legionella* contamination and amoebae has been reported for numerous sources, including hot springs. Waters from two hot spring recreation complexes in central and southern Taiwan demonstrated a positive correlation with *H. vermiformis* presence [35]. Molecular methods detected contamination in 48% of samples with an unusually high minimum concentration of 14 GU/mL. The lack of correlation between the occurrence of *Legionella* and *Acanthamoeba* or *Naegleria* species suggests a host preference for *Hartmannella*. 

Recreational hot springs continue to be an increasingly relevant and studied source of *Legionella* occurrence and transmission. In Taiwan, 45 of 48 similar hot springs tested positive for *Legionella* via PCR [58], demonstrating the extremely common contamination that is possible for such sites. Additionally, the identification of a novel species (*L. thermalis*) that was detected in recreational springs highlights the importance of examination for these and other commonly over-looked sources [59].

Unsurprisingly, *Legionella* spp. from differing environments tend to be genetically distant, yet still may show a wide diversity. A sampling of hot springs and engineered water systems from a relatively geographically linked area around Wenzhou, China, produced 13 strains from 4 different serogroups [60]. Hot springs tended to have higher positivity (62.5%) and concentrations (up to 107 CFU/mL). Additionally, strains from hot springs tended to have more genetic homology than those from cooling towers or premise plumbing samples.

## 9. Saltwater

Aquatic environments with low to moderate osmotic pressures are thought to be the primary aquatic natural habitats for *Legionella*. While saltwater may produce environmental stress for bacterial cells, natural occurrence in and tolerance to this medium has long been reported for *Legionella* [62,63]. Research that is focused on the ecology of *Legionella* in saline sources is limited, often assuming freshwater contamination. Although this may often be the case, reports in high osmolality, isolated, and oceanic sites indicate salty environments may also be a natural habitat for *Legionella*. The summarized results from studies that are discussed in this section are listed in Table 3.

## 10. Marine

With the potential for exposure from recreational waters, as well as the risk that is posed by contamination of seawater sources for desalination plants, contamination of marine waters with *Legionella* has the capability to serve as a threat to public health. In one of the first reports of *Legionella* in saline waters, multiple coastal and estuarine sites in Puerto Rico demonstrated contamination [64]. The occurrence was detected in all 26 sites from 5 geographically separated areas on the island that were examined. Although several species were reported, *L. pneumophila*, including serogroup 1, was by far the most commonly detected at high concentrations of 3.1 × 10^4^ CFU/mL. Environmental pollution from storm runoff, sewage, and factory effluent (including a large rum distillery) were presumed to play a role in the high levels of contamination that were recorded.

During examination of the impact of treated sewage on the presence of *Legionella* in ocean-receiving waters, contamination was detected both at outfalls and coastal waters in California, USA [65]. Samples from surface water and 30 m deep at outfall locations, as well as from a nearby surf zone were collected. The contamination was clearly traced from the deep-water outfalls, with positivity and concentrations dropping from 75% and 10^3^ cells/mL to 14% and 28 cells/mL in the surf zone. A lack of culture results and seed experiments in ocean water samples suggested the formation of viable but non-culturable cells in response to the saltwater environment.

The tolerance of *Legionella* to saltwater compared to other naturally occurring microbes has been demonstrated. In a mesocosm study performed on 300 L of seawater from the Gulf of Lyons in the Mediterranean Sea, *Legionella* increased in abundance over time [66] After 281 h in the confined space of the tank, drastic changes in the microbial community of the collected seawater were observed with *Legionella* comprising nearly 20% of the bacterial clones. Present in the original sample, this recorded increase suggests the potential for blooms of this pathogen under certain conditions.

Anthropogenic pollution is often implicated in the occurrence of *Legionella* in marine environments. Examination of water samples from an estuarine region in the southern coast of Sao Paulo state, Brazil, revealed multiple species, including *L. pneumophila* [20]. The lack of diversity upstream of the site affected by untreated domestic sewage led to the conclusion that the salinity (7.8%) and human pollution of the estuary may have created environmental conditions favoring multiple species. 

In a microbial ecology study on bacterial communities that were associated with healthy and diseased corals, *Legionella* were detected in samples containing coral and seawater [67]. Multiple species of corals were examined from sites along the coast of Taiwan, with *Legionella* DNA being detected in association with both healthy and sick specimens. The possibility of transient microbes from nearby anthropogenic sources, including nuclear power plant discharge, was suggested.

*Legionella* were detected in a warm-water coastal lagoon off the southern Adriatic coast of Italy [72]. While examining microbial communities during jellyfish blooms, molecular methods detected a previously unidentified species in surface samples that were collected in a site with low jellyfish levels. While a slightly cold freshwater spring intrusion may have led to the occurrence that was reported, the <28 °C waters could have facilitated growth in this environment.

Global warming may potentially lead to increasing water temperatures that are sufficient to support *Legionella* growth in new environments. In an ecological study to determine the effects of coastal water warming, *Legionella* occurrence was reported in water samples from Suruga Bay in Japan [73]. Multiple species were isolated from immediately processed samples that were collected in the fall and incubation at elevated temperature was shown to drastically increase the detected diversity in one sample.

The potential impact of anthropogenic pollution *Legionella* populations has been demonstrated in both freshwater and marine environments. In a survey of a Chesapeake Bay inlet, 38/38 samples near a wastewater treatment plant tested positive for *Legionella* genetic markers, with GU/mL levels in the thousands, increasing following rain events [74]. Similarly, field sampling in the Virgin Islands proceeding a hurricane reported higher concentrations of *Legionella* in more polluted coastal water sites [75]. Confusingly, temperature was strongly correlated with *Legionella* contamination in the Chesapeake Bay but not the Virgin Islands.

## 11. Inland Sources

While less common than ocean sources, inland saline aquatic environments share many characteristics, including the potential for human exposure. Amoeba isolates from 8/8 sediment samples from sewage-contaminated areas of the Great Salt Lake, Utah, United States contained *Legionella* DNA [68]. The sampling sites varied in salinity (3–140%) and were either mesotrophic or hypereutrophic. A total of 74% of the 53 amoeba isolates tested positive and *L. pneumophila* was an uncommon species that was detected, only present in 7.5% of amoebae. Samples that were collected in June showed increased positivity compared to those from August, potentially due to increased temperatures. 

It can be assumed unique *Legionella* species that are naturally inhabiting saline environments would be adapted to their habitat, potentially with distinct proteomic profiles. A previously unidentified species, *L. tunisiensis*, was isolated via amoeba co-culturing in Lake Sabka, a hypersaline lake in Tunisia [69]. This novel species is interesting due to its large amount of potential coding sequences and unusually high number of resistance genes (50% more sequences and 37 more genes than *L. pneumophila* 130b). While not determined, a large number of encoded proteins could be related to mechanisms for overcoming osmotic stress. 

Perhaps the most likely source of transmission of *Legionella* in saltwater, saline baths have demonstrated contamination. *L. pneumophila* was detected in 100% (15/15) of water and floating biofilm samples from balneotherapy facilities in Poland [70]. Water for the baths was supplied by thermal saline groundwater sources 700–1700 m deep and were measured at 30.7–36.5 °C. *Legionella* were major members of the microbial community, constituting 22% of the bacteria and reaching concentrations of 10^5^ cells/mL. The combination of ideal growth temperatures, age of facilities (up to 80 years old), and low salinities of 1.5–5% likely contributed to the high levels of occurrence. 

Another unique saline environment that was found to contain *Legionella* is Hot Lake, Washington, United States [71]. Metagenomic analysis of the bacterial community of the lake margin soil and water samples from this epsomite lake with high levels of magnesium sulfate revealed a high relative abundance of *Legionella* clones. While the samples appeared to contain similar microbiomes to common soil, with actinomycetes dominating by a wide margin, *Legionella* was one of the next most common genera that were detected. Even without cultured isolates, these results demonstrate that high representation of *Legionella* is possible in waters with near-saturated salinities. 

With the majority of life on the planet residing in the ocean, it should come as no surprise that saltwater can be a rich environment with an incredibly important microbiome for ecological and public health purposes [76], possessing nutrients and conditions that are necessary for *Legionella* persistence and growth. While occurrence may be lower than other sources, saline aquatic habitats still serve as an important niche for *Legionella* both from an environmental reservoir and potential source of direct human exposure.

## 12. Drinking Water

It is thought that a large proportion of legionellosis cases can be linked to an exposure route via cooling towers or in-premise plumbing water, with these sources of transmission accounting for a majority of reported incidence in many parts of the world [2,10]. The importance of point-of-use distributed water signifies that the water treatment plants and distribution systems delivering this water are also important in the transmission of legionellosis. While it is often assumed that appropriate drinking water treatment sufficient to reduce gastrointestinal pathogen concentrations to safe levels will do the same for *Legionella* [77], this may not necessarily be true given their ability to resist environmental stress and replicate in oligotrophic conditions; there are key differences separating them from other waterborne pathogens and highlighting the importance of understanding the complex dynamics of populations in drinking water systems and treatment plants. A Morbidity and Mortality Weekly Report demonstrating *Legionella* were responsible for 66% of waterborne-disease outbreaks and 26% of illnesses suggests that current standards of drinking-water treatment may not be sufficient [78]. The summarized results from the studies that are discussed in this section are listed in Table 4.

## 13. Drinking Water Treatment Plants

While early investigations on the presence of *Legionella* within drinking water treatment plants frequently produced negative results [93] perhaps due to improved detection methodology, later studies have been successful in isolation from this environment. A molecular ecology study on bacterial biofilm communities in a drinking water production system fed by the Rhine River, identified the presence of *Legionella* DNA [80].

Analysis of amoebae-resistant bacteria after water treatment steps in a plant that was supplied by surface water in Paris, France, revealed the presence of *Legionella* within the isolated amoebae [82]. While only one *Legionella*-like amoeba pathogen was successfully cultured, indigenous amoeba isolates from sand biofilm, carbon filter biofilm, and post-carbon filtered water contained DNA from multiple *Legionella* species. The increased detection of *Legionella* within hosts suggests a potential for increased survival throughout the water treatment processes via amoeba endoparasitization.

The amplification of *Legionella* during water treatment may be possible, particularly in biologically active steps or with processing of anaerobic source water. A drinking water treatment plant in the Netherlands that was supplied by anaerobic groundwater demonstrated drastic increases in *Legionella* contamination during treatment [52]. While raw water and aeration tanks samples were below the limit of detection, rapid sand filter, pellet softener, and treated water samples each contained approximately 10 cells/mL. Although no isolates were cultured, the presence of multiple species, including *L. pneumophila*, after treatment presents a counter-intuitive dynamic in the system that was examined.

Another example of increase in *Legionella* concentration during water treatment was demonstrated in samples that were collected from a chlorination-based plant in Hubei province, China [31]. Molecular analysis of biofilm and water samples from a reaction tank, settling pond, sand filter, and clear water tank revealed a constant presence of *Legionella* in every sample type, albeit with fluctuating levels throughout the treatment process. Surprisingly, while the concentrations decreased significantly in clear water tank water samples, biofilm from this source contained over an order higher number of cells than any other sample type, 3 × 10^3^ GU/g. While the literal concentration of *Legionella* may have led to these results, growth in these biologically active systems’ biofilms would be a reasonable possibility.

## 14. Drinking Water Distribution Systems

With the presence of *Legionella* within drinking water treatment plants well documented, it should come as no surprise that frequent occurrence has been demonstrated within drinking water distribution systems as well. In an environmental study on biofilms within the distribution system of a town that is fed by the Rhine River [79], *Legionella* were detected within biofilm that formed on a variety of coupons that were placed along points downstream of a treatment plant. While culturable cells were not observed, a high positivity by molecular detection was recorded, with 87.5% (14/16) of the sampling sets positive. Chlorine residual seemed to have little impact on *Legionella* survival in these systems as high positivity was reported both before and after the plant shifted from chlorine dioxide to UV based treatment.

The loss of disinfectant residual through the length of a distribution system can lead to water quality issues, including increased *Legionella* levels. Investigation of a distribution system in Pinellas County, FL, United States, revealed common contamination [81]. While *Legionella* presence was observed in biofilm and water samples from multiple sites along the system, including sampling stations, backflow valves, and master meters, dead-end streets had higher positivity. Additional sampling of previously identified high risk sites, including the ends of the distribution network and backwash areas, produce a single sampling event with 20% positivity, as well. The denaturing gradient gel electrophoresis analysis of certain biofilm samples showed *Legionella* to dominate populations, suggesting that low levels of chlorine residual may have had a greater impact on other, less resilient microbes.

Similar to the results from water treatment plants, water quality parameters often appear to have a seemingly minor impact on *Legionella* populations within drinking water distribution systems. Samples that were collected over time from sites within a Hungarian distribution system examined the relationships between pathogen occurrence and a number of environmental factors [83]. Little to no correlation was measured between contamination and temperature, turbidity, nitrate, sulfate, heterotrophic plate counts, total organic carbon, or chemical oxygen demand. *Legionella* were detected more commonly than *Pseudomonas aeruginosa*, a common member of the microbial community in drinking water.

In contrast to other reports, an environmental study on drinking water supplies in the Caribbean observed a correlation between *Legionella* concentration and certain environmental factors [84]. A total of three distribution systems that were connected to reverse osmosis saltwater treatment plants with either UV or chlorine disinfection were examined. An occasional correlation was measured between *Legionella* concentration and turbidity, ATP, and *H. vermiformis* levels. Culturable cells were detected in 41/49 samples at distances up to 15 km from treatment, with *L. pneumophila* representing 80% of colonies that were observed in samples from the UV treated systems. While positivity was high with both forms of treatment, concentrations were significantly lower in the chlorinated system.

Certain regions have recently made shifts toward using chloramination for drinking water disinfection in place of chlorination, and while chloramine has been proven effective against *Legionella*, in-depth examination of chloraminated systems are rare. A large-scale molecular survey of opportunistic pathogens and amoebae in two recently chloraminated drinking water distribution systems in the United States demonstrated high levels of *Legionella* contamination in taps that were connected to the system [39]. *Legionella* was detected in >33% (30/90) samples from one system and >83% (45/54) from another, with *L. pneumophila* observed in 4.4% and 5%, respectively. While high concentrations greater than 10^3^ GU/mL were occasionally observed, culturable cells were only present in a single sample that was collected, suggesting a high potential for chloramine to trigger viable but non-culturable *Legionella*.

In another study of distribution systems, *Legionella* contamination rates from rural areas in eastern Poland were low [53]. Using a combination of culture-based and molecular detection methodology, only 7.4% of 27 samples that were collected from cold water taps on farms in three villages receiving chlorinated groundwater were positive. Although collected from hot water sources, the samples that were collected from the city of Lublin, a more urban environment, were significantly more likely to contain *Legionella*, with a 49% (22/45) positivity.

In certain regions with poorly established civil infrastructure or widespread populations, water quality issues may arise, and non-conventional drinking water distribution systems are often implemented, presenting potential ramifications for *Legionella* occurrence. Drinking water distribution systems and reverse osmosis water supply tankers in Basra Governate, Iraq, were found to contain high levels of contamination [86]. A total of 100% (18/18) of the samples that were collected from distribution system sites contained *Legionella* concentrations up to 400 CFU/mL, and while tankers exhibited lower levels of contamination, >66% (6/19) were positive. Drinking water treatment plant deficiencies may have played a role as a majority of effluent samples from 13 plants contained *Legionella* with a high concentration of 1.8 × 10^3^ CFU/mL recorded. Indicative of additional public health risk, over 77% of *L. pneumophila* isolates that were identified in the study belonged to serogroup 1.

Individual studies examining drinking water systems from large regions have rarely been performed, despite the fact that stark variations have been reported with different detection methodologies and between systems. In one of the only such studies that was conducted in the United States, *L. pneumophila* serogroup 1 was commonly detected in cold water taps from 40 geographically dispersed sites [87]. A total of 272 samples from 68 sites were analyzed by PCR, revealing 29% of samples and 47% of sites were positive and an average concentration of 2 GU/mL. While similar detection rates and concentrations were observed for water containing chlorine or monochloramine, the variability in concentration was much lower for the latter.

Seasonality of *Legionella* in certain sources is feasible due to differences in temperatures situationally, allowing substantial growth potential. This effect was observed in both a chlorinated and a chloraminated water distribution system in South Australia [88]. *Legionella* and *L. pneumophila* concentrations were highest in both systems in the summer, with exceptionally high *Legionella* concentrations of up to 10^6^ and 10^3^ GU/mL, respectively. Levels were also noted to increase in a dead end and with increasing distance along one of the systems. While the warm summer water temperatures up to 27 °C may have contributed to the observed results due to reduction in disinfectant residual, increased potential for growth may have also been a factor.

Extensive differences in *Legionella* contamination may exist within relatively similar distribution systems. Biofilms that were sampled from domestic water meters in two close-by networks that were located in central Arizona, United States, exhibited vastly differing positivity [90]. Molecular analysis on biofilms that were collected from 67 water meters originating from the two distribution systems showed *Legionella* positivity of 26% in one (14% for *L. pneumophila*), with no positive samples coming from the other. Such a stark difference was surprising, given the nearly identical source waters and largely similar treatment methods that were employed for the two systems.

Drinking water distribution system infrastructure can often play a role in pathogen contamination, with larger municipal systems being more prone to contain areas of stagnation. High-risk areas in distribution networks, notably dead ends, have been associated with increased potential for water-borne pathogens due to several possible factors, including stagnation and loss of residual disinfectants. A phylogenetic study on *L. pneumophila* in biofilm and water samples that were collected from a variety of points, including suspect areas in the city of Alcoy, Spain, revealed a significant increase in positivity in dead ends [89]. Out of 180 chlorinated or unchlorinated samples that were collected, 18 were positive for *Legionella*, including 12/18 dead ends that were sampled. In addition, the only culturable *Legionella* that were detected in the study were isolated from street dead end samples. Interestingly, a positive correlation was measured with temperature and contamination but not chlorine presence. The greatly increased positivity in select locations highlights the importance of proper sampling to gauge the true levels of contamination in drinking water distribution systems.

Residual disinfection is assumed to lower total levels of pathogens in drinking water distributions systems and a comparison between a chloraminated system in the US versus a no-residual system in Norway demonstrated this held true for *Legionella* [91]. While no *Legionella* were detected in biofilm from water mains in the chloraminated system, 10/23 samples from a residual-free system had concentrations as high as 7.8 × 10^4^ gene copies/cm^2^. Tap-water that was collected throughout both systems, however, showed relatively similar occurrence and concentrations, highlighting the often-reported effectiveness of chloramine on biofilm microbial populations.

Wide-spread sampling regimes in distribution systems often reveal ubiquitous *Legionella* presence in water mains. In a broad sampling of four drinking water distribution systems in Paris, *Legionella* were detected in 192/268 water samples at concentrations greater than 100 GU/mL [92]. While no correlation was reported between the contamination and a series of water quality parameters, including temperature, a high degree of seasonality was observed with higher concentrations and more consistent detection of *Legionella* in the spring.

Due to studies reporting low concentrations and sporadic occurrence, the relevance of *Legionella* contamination within drinking water distribution systems is typically overshadowed by that in buildings and cooling towers. Dismissal of the importance of distribution systems in legionellosis transmission could be unwarranted as increases in *Legionella* contamination within them due to disruptions, treatment failures, or other events and factors impacting the distributed water quality may play a part in outbreaks [2,94]. While total elimination of *Legionella* in drinking water systems may not be feasible, occurrence data in tap-water will be needed to develop reasonably specific treatment and monitoring guidelines in this source.

## 15. Conclusions

With increasing incidence of legionellosis worldwide and novel sources of transmission continuing to be discovered, the importance of documenting all possible reservoirs for *Legionella* is clear. This importance extends to environmental habitats that are not commonly linked to human disease at the current time. Additional knowledge on the occurrence of these pathogens in source water and distribution networks will be critical for the development of effective practices to limit transmission via commonly implicated point sources. Investigation of these poorly understood sources will also provide fundamental insight that is relevant to the physiology and ecology of *Legionella* in all environments, including cooling towers and premise plumbing systems, providing additional tools to combat legionellosis.

A longstanding and current limitation of all such investigations concerns the detection methodology that is employed. As demonstrated throughout this review, differing methods are capable of delivering contrasting results, both for detection and quantification. In addition to future studies that are designed to help researchers better interpret the ‘meaning’ of current detection techniques beyond raw data, research that is focused on improving current or developing novel methods will be critical for both field and laboratory studies of *Legionella*. Examples of several such advances include use of immunofluorescence solid phase cytometry [28] and microcolony-based quantification [85], both of which may help overcome error that is associated with environmental samples containing PCR inhibitors and poorly culturable cells.

## Figures and Tables

**Table 1:** Occurrence of *Legionella* in Surface Freshwater.

Geographic Location	% Positivity	Concentration	Reference
USA: NC, SC, GA, FL, AL, IN, IL	DFA: 99.5% samples, 98.5% sites	9.1–3.3 × 10^4^ cells/mL	[17]
USA: CA	DFA: 100%, PCR: 100%, Cul.: 25%	DFA: <0.1–>0.1 cells/mL PCR: <10^3^–10^3^ cells/mL	[18]
Netherlands	PCR: 100%	20–2.5 × 10^3^ cells/mL	[19]
Itanhaém River, Sao Paolo, Brazil	Cul.: 0%, PCR: 100%	N/A	[20]
Lake Pontchartrain, New Orleans, USA	PCR: 72.9% samples, 100% sites	N/A	[21]
Antarctica (King George Island)	PCR: 100%, Cul.: 50%	0.02 CFU/mL	[22]
Auckland, New Zealand	Cul.: 15% *Lp*	0.3–8 × 10^2^ CFU/mL	[23]
Tokyo, Japan	Cul.: 25%. PCR: 60%	<0.2 CFU/mL–>10 CFU/mL	[24]
Elbe River, Dresden, Germany	NA	N/A	[25]
Glomma River, Norway	Cul.: 42.3% samples, 87.5% sites	40–1.9 × 10^3^ CFU/mL	[26]
Tech River, France	Cul.: 20.8%, PCR: 100%	0.05–0.583 CFU/mL. 7.39–936 GU/mL	[27]
Pyranees, France	Cul: 20.8%. PCR: 100%	Cul.: 0.19–0.22 CFU/mL PCR: 1.1–2 × 10^2^ cells/mL	[28]
Lake Taihu, China	PCR: 65.6%	NA	[29]
Jiulong River, Fujian province, China	PCR: 100%	<5 × 10^2^–2.5 × 10^4^ GU/mL	[30]
Hubei Province, China	PCR: 100%	Biofilm:10–10^3^ GU/g. Water: 30–100 GU/mL	[31]
Queensland, Australia	PCR: 6%	16–100 GU/mL	[32]
East Cape Province, South Africa	PCR: 86%	N/A	[33]
Utrecht, Netherlands	Cul.: 3.9%	N/A	[34]
Puzih river, Taiwan	PCR: 63.1% *Leg*:, 7.7% *Lp*	18–10^3^ GU/mL	[35]
Taiwan	PCR: 35.5% samples, 78.9% reservoirs	0.05–1.6 × 10^6^ cells/mL	[36]
South Korea	PCR: 100% sites, 14% samples	N/A	[37]
Antarctica	PCR: 36.8%	N/A	[38]

DFA: direct fluorescent antibody microscopy, Cul.: cultivation techniques, N/A: no data available, *Lp*: *Legionella pneumophila*, *Leg*: *Legionella* spp.

**Table 2:** Occurrence of *Legionella* in Ground Freshwater.

Geographic Location	% Positivity	Concentration	Reference
Savoie, France	Cul: NA	1–100 CFU/mL	[47]
USA: AL, FL, ID, IL, IN, MD, MI, MN, MT, NY, NC, OH, OR, TX, VT, WA, USA	PCR: 94.8%, Cul.: 7%	<44–>44 cells/mL	[18]
US	Cul.: 100%	Water: 0.1–840 CFU/mL. Biofilm: 2–267 CFU/cm^2^	[48]
USA: FL, AZ, TX, IL, MO, MI, NJ. Canada: Ontario, New Brunswick	Cul.: 33.3%. PCR: 24.1%. Combined: 46% (58% water, 34.1% biofilm)	Cul: Water: 0.1–100 CFU/mL, Biofilm: 3–1.2 × 10^2^ CFU/cm^2^	[49]
Central Portugal	Cul.: Water: 58.6%, Well: 83.3%, Biofilm: 100%	Water: 0.05–400 CFU/mL. Biofilm: 24–240 CFU/mL	[50]
The Netherlands	PCR: Anaerobic Water: 42.9%, Aerobic Water: 88.9%	Anaerobic: <0.2–2.4 cells/mL Aerobic: <0.2–25 cells/mL	[19]
Taiwan	Cul.: Spring water: 33.3%, Hot tub/spa water: 33.3%	N/A	[51]
Netherlands	PCR: 60%, Cul.: 0%	PCR: 0.076–0.39 cells/mL	[52]
Southern Taiwan	Cul. and PCR: 38%	N/A	[40]
Eastern Poland	Cul.: 6.3%, PCR: 62.5%	N/A	[53]
Japan	Cul.: 37.2% samples, 72.7% prefectures	0.1–3 CFU/mL	[54]
Northern Tunisia	Cul.: 22%. PCR: 70.1%	Cul.: 0.1–8.2 CFU/mL. PCR: 0.1–420 GU/mL	[55]
Beijing, China	Cul.: 74.4%. PCR: 100%. EMA qPCR: 100%	Cul.: 0.1–216 CFU/mL. PCR: 1.47–1557.75 GU/mL. EMA qPCR: 0.2–301.69 GU/mL	[56]
Central and Southern Taiwan	PCR: 47.5% *Leg*, 9.8% *Lp*	14–170 GU/mL	[35]
Kathmandu Valley, Nepal	PCR: 73%	N/A	[57]
Taiwan	Cul.: 93.8%	Cul.: 72.1–5.7 × 10^6^ CFU/mL	[58]
Tokyo, Japan	Cul.: N/A	Cul.: N/A	[59]
Wenzhou, China	PCR: 62.5%	Cul: 0.2–107 CFU/mL	[60]
Apulia Region, Italy	Cul: 21.2%, PCR: 32.4%	PCR: 0.263–2.87 × 10^3^ GU/mL Cul: 50 CFU/mL (maximum)	[61]

Cul.: cultivation techniques, N/A: no data available, SD: standard deviation, EMA qPCR: ethidium monoazide quantitative PCR, *Lp*: *Legionella pneumophila*, *Leg*: *Legionella* spp.

**Table 3:** Occurrence of *Legionella* in Saltwater.

Geographic Location	% Positivity	Concentration	Reference
Puerto Rico	DFA: 100%	*Leg*: 8.67 × 10^3^–5.6 × 10^4^ cells/mL, *Lp*: 2.1 × 10^3^–3.1 × 10^4^ cells/mL	[64]
USA	Cul.: 0%, PCR: 30%, DFA: 26.7%	PCR: <10^3^–>10^3^ cells/mL. DFA: 4–28 cells/mL	[65]
Gulf of Lyons, Mediterranean Sea	PCR: 100%	N/A	[66]
Itanhaém River, Sao Paolo, Brazil	Cul.: 0%, PCR: 100%	N/A	[20]
3 coral reef sites; Southern Taiwan	PCR: N/A	N/A	[67]
Mt Hope Bay, New England, and Great Salt Lake, UT, USA	PCR: 88.6% soil samples	N/A	[68]
Lake Sabka, Tunisia	Cul.: N/A	N/A	[69]
Poland	DFA: 100%	*Leg*: 1.98 × 10^3^–3.2 × 10^4^ cells/mL, *Lp*: 70–4.85 × 10^3^ cells/mL	[70]
Hot Lake, WA, USA	PCR: N/A	N/A	[71]
Varano Lagoon, Adriatic coast. Apulia, Italy	PCR: 50% sampling sites	N/A	[72]
Suruga Bay, Japan. Ha Long Bay, Vietnam	PCR: ≥30%	N/A	[73]
Oxford, Maryland, USA	PCR: 100%	>10^3^ GU/mL (maximum)	[74]
St. Thomas, Virgin Islands	PCR: 10%	N/A	[75]

DFA: direct fluorescent antibody microscopy, *Leg*: *Legionella* spp., *Lp*: *Legionella pneumophila*, Cul.: cultivation techniques, N/A: no data available.

**Table 4:** Occurrence of *Legionella* in Distribution System Drinking Water.

Geographic Location	% Positivity	Concentration	Reference
Central Europe	PCR: 100% platelet materials, 87.5% sampling sets	N/A	[79]
Central Europe	PCR: 100%	N/A	[80]
Pinellas County, Florida, USA	PCR: 20% of select sites	N/A	[81]
Drinking Water Treatment Plant Paris, France.	PCR: 29% samples	N/A	[82]
Hungary	PCR: 40% (Post chlorination 8%)	N/A	[83]
Leeward Antilles, Caribbean Sea	PCR: 84%	UV: 0.3–250 CFU/mL, Cl: <0.25–65 CFU/mL	[84]
Netherlands	Cul.: 0%. PCR: 100% water, 93% biofilm	Water: 0.13–5.7 cells/mL, biofilm: 1.8–390 cells/cm^2^	[52]
Japan	Cul.: 17%, MC-FA 28%	Cul.: 0.1–12 CFU/mL. MC-FA: 0.02–19 micro CFU/mL	[85]
Eastern Poland	Cul.: 0%. PCR: 7.4%	N/A	[53]
USA: FL and VA	PCR: Water: 51.7% (4.8% *Lp*), Biofilm: 34.6% (3.8% *Lp*)	*Leg:* 2.3 × 10^3^ GU/mL water, 1.5 × 10^6^ /swab biofilm. *Lp*: 219.4 GU/mL water, 1.9 × 10^4^ GU/swab biofilm	[39]
Basra, Iraq	Cul.: WTP: 70%, DS: 100% sites, Water tankers: 31.6%.	WTP: 10–5.6 × 10^4^ CFU/mL, DS: 20–400 CFU/mL	[86]
Hubei Province, China	PCR: 100% sampling sites	Biofilm: 10–3000 GU/g, Water: 0.316–10 GU/mL	[31]
USA. 25 states	PCR: 20% samples, 47% taps	0.04–3.65 × 10^2^ GU/mL	[87]
South Australia, Australia	PCR: 100% sampling sites	Cl: *Leg:* 3–1238 GU/mL, *Lp*: 3–1981 GU/mL. Cla: *Leg:* 24–316,956 GU/mL, *Lp*: 3–3176 GU/mL	[88]
Alcoy, Spain	Cul.: 7.5% water, 21.6% biofilm	<0.04–0.45 CFU/mL	[89]
Arizona, USA	Cul.: 0%. PCR: *Leg*: 13.4%, *Lp*: 7.5%	N/A	[90]
US and Norway	PCR. biofilm: 0% cla, 43.47% no residual system	Biofilm: 7.8 × 10^4^ GU/ cm^2^ (maximum)	[91]
Paris, France	PCR: 52.17% sampling sites	>10^2^ GU/mL (maximum)	[92]

N/A: no data available, UV: ultraviolet light disinfection, Cl: chlorine disinfection, Cul.: cultivation techniques, MC-FA: micro-colony fluorescent antibody microscopy, *Lp*: *Legionella pneumophila*, *Leg*: *Legionella* spp., WTP: water treatment plant, DS: distribution system, Cla: chloramine disinfection.

## Data Availability

Not applicable.
